# The problem with calling it “unexplained infertility”

**DOI:** 10.1016/j.xfre.2025.12.007

**Published:** 2026-01-05

**Authors:** Richard J. Paulson

**Affiliations:** USC-HRC Fertility, Division of Reproductive Endocrinology and Infertility, Department of Obstetrics and Gynecology, Keck School of Medicine, University of Southern California, Los Angeles, California

A couple came to see me for a second opinion last week. They had been attempting conception for about three years and had sought the help of a local fertility specialist. Their workup was normal, and they were told that they had “unexplained infertility.” Because they had been trying for more than two years, the physician recommended in vitro fertilization (IVF) as the next step in their fertility treatment.

The couple explained that the reason for seeking a second opinion was confusion. If the physician could not determine the cause of their infertility, how could they, in good conscience, recommend an expensive and invasive procedure that could potentially fail for the same unexplained reason? In their words, *“How can we move on to IVF if we don’t even understand why we are infertile in the first place?”*

I did my best to clarify that the term “unexplained infertility” is simply a designation for a couple with a normal evaluation who is not achieving conception via correctly timed intercourse (1). The term dates back to the era when infertility was first classified into general categories such as “male factor,” “ovulatory factor,” or “pelvic factor.” Those who did not meet diagnostic criteria for one of those designations were assigned the label “unexplained.” Although the classification “unexplained infertility” is widely accepted (2), it is antiquated, because we do, in fact, have a very good understanding of what is causing the lack of conception.

Although there are very rare cases of unexplained oocyte maturation failure or unexplained defects in sperm function, the vast majority of so-called unexplained infertility represents a mechanical problem that prevents the egg and sperm from successfully coming together. We know this because when this mechanical impediment is overcome, conception can occur just as it does in couples without infertility. Ovarian stimulation with intrauterine insemination (IUI) brings sperm closer to the site of fertilization and does increase the chance of conception. However, the most effective approach to treating unexplained infertility is collecting eggs and sperm and physically bringing them together.

Today, this is most commonly accomplished through IVF. In the early days of assisted reproduction, however, a procedure called gamete intrafallopian transfer (GIFT) was frequently used for unexplained infertility (3, 4). As with IVF, GIFT required the collection of eggs and sperm. Unlike IVF, fertilization did not occur in the laboratory. Instead, egg and sperm were placed laparoscopically into the distal portion of the fallopian tube in a small amount of culture medium. Success rates with GIFT were much higher than with IUI, providing strong evidence that the barrier to conception had simply been mechanical. There was no intrinsic problem with egg quality, sperm fertilizing capacity, or the ability of the uterus to sustain pregnancy.

In current practice, couples with a normal infertility evaluation are treated with IVF, which bypasses the mechanical impediment to gamete interaction and offers the highest probability of conception. Proceeding with IVF is therefore a very reasonable course of action, and couples have every reason to expect the standard success rates associated with IVF treatment.

This patient encounter reminded me of the problems inherent in the continued use of the term “unexplained infertility.” What patients hear is “we don’t know,” or worse, “we can’t figure out what is wrong, and something rare or serious may be causing your infertility.” My patients clearly believed that something was fundamentally wrong, which made them reluctant to pursue IVF. This misunderstanding alone should compel us to abandon this imprecise and antiquated designation.

Perhaps we have retained the term “unexplained infertility” because there is no obvious alternative. What should replace it? “Infertility with a normal workup” would at least be accurate. “Infertility with no specific origin” or “infertility beyond current diagnostic resolution” is less satisfying because the underlying issue appears to be mechanical rather than unknowable. “Unascertained infertility” is concise but functionally indistinguishable from “unexplained.” Perhaps, patients might be most comfortable with “idiopathic infertility,” because “idiopathic” is commonly used for many medical conditions without a single identifiable cause.

The bottom line is that continued use of the diagnosis “unexplained infertility” is a disservice to our patients. The term is unclear, creates confusion, and generates unnecessary anxiety. It is also inaccurate and inconsistent with our current understanding of reproductive physiology. Professional societies should come together to develop a more accurate and patient-centered term that we can all adopt. Because this is simply a matter of nomenclature, a full Delphi consensus process should not be necessary (5). What is necessary is a willingness to change and a commitment to clarity. Our patients deserve better than “unexplained infertility.”

## Declaration of Generative AI and AI-Assisted Technologies in the Writing Process

During the preparation of this work, the author used ChatGPT to ensure the accuracy of grammar and spelling. After using this service, the author reviewed and edited the content as needed and takes full responsibility for the content of the published article.

## Declaration of Interests

R.J.P. has nothing to disclose.
